# Effect of combining glucocorticoids with Compound A on glucocorticoid receptor responsiveness in lymphoid malignancies

**DOI:** 10.1371/journal.pone.0197000

**Published:** 2018-05-08

**Authors:** Dorien Clarisse, Karlien Van Wesemael, Jan Tavernier, Fritz Offner, Ilse M. Beck, Karolien De Bosscher

**Affiliations:** 1 Receptor Research Laboratories, Nuclear Receptor Lab (NRL) and Cytokine Receptor Lab (CRL), Department for Biomolecular Medicine, VIB-UGent Center for Medical Biotechnology, Ghent University, Ghent, Belgium; 2 Laboratory of Experimental Cancer Research (LECR), Department of Radiation Oncology and Experimental Cancer Research, Ghent University, Ghent, Belgium; 3 Cancer Research Institute Ghent (CRIG), Ghent, Belgium; 4 Hematology, Department of Internal Medicine, Ghent University Hospital, Ghent, Belgium; 5 Department of Health Sciences, Odisee University College, Ghent, Belgium; Wayne State University, UNITED STATES

## Abstract

Glucocorticoids (GCs) are a cornerstone in the treatment of lymphoid malignancies such as multiple myeloma (MM) and acute lymphoblastic leukemia (ALL). Yet, prolonged GC use is hampered by deleterious GC-related side effects and the emergence of GC resistance. To tackle and overcome these GC-related problems, the applicability of selective glucocorticoid receptor agonists and modulators was studied, in search of fewer side-effects and at least equal therapeutic efficacy as classic GCs. Compound A (CpdA) is a prototypical example of such a selective glucocorticoid receptor modulator and does not support GR-mediated transactivation. Here, we examined whether the combination of CpdA with the classic GC dexamethasone (Dex) may improve GC responsiveness of MM and ALL cell lines. We find that the combination of Dex and CpdA does not substantially enhance GC-mediated cell killing. In line, several apoptosis hallmarks, such as caspase 3/7 activity, PARP cleavage and the levels of cleaved-caspase 3 remain unchanged upon combining Dex with CpdA. Moreover, we monitor no additional inhibition of cell proliferation and the homologous downregulation of GR is not counteracted by the combination of Dex and CpdA. In addition, CpdA is unable to modulate Dex-liganded GR transactivation and transrepression, yet, Dex-mediated transrepression is also aberrant in these lymphoid cell lines. Together, transrepression-favoring compounds, alone or combined with GCs, do not seem a valid strategy in the treatment of lymphoid malignancies.

## Introduction

Endogenous glucocorticoids (GCs), e.g. cortisol in humans, are stress-stimulated steroidal hormones that modulate metabolism, inflammation, development, reproduction and the immune system [1,2]. Therapeutically, exogenous GCs, e.g. dexamethasone (Dex), are mostly used to treat inflammatory disorders such as rheumatoid arthritis, inflammatory bowel disease, asthma and atopic dermatitis [3]. In addition, GCs are deployed in cancer, either as adjuvant (e.g. breast cancer) or as anti-cancer therapy (e.g. multiple myeloma) [4]. As co-medication, GCs reduce edema, nausea and vomiting, avoid uncontrolled immune reactions caused by chemotherapeutics and alleviate pain [5]. In lymphoid malignancies, such as multiple myeloma (MM) [6] and acute lymphoblastic leukemia (ALL) [7], GCs induce apoptosis of the malignant cells [5,8].

At the molecular level, GCs bind to the glucocorticoid receptor (GR), a transcription factor and nuclear receptor. GR’s genomic mechanisms have long been divided into two main modes, i.e. transactivation, which promotes the transcription of glucocorticoid responsive element (GRE)-driven genes (e.g. *GILZ*), and transrepression, via which GR inhibits the expression of genes mediated by transcription factors such as NF-κB and AP-1 [9]. Yet, the underlying interaction modes of GR with DNA, as a monomer or dimer, and with other transcription factors, remain a subject of discussion [10,11]. Non-genomic mechanisms include, among other mechanisms, translocation of GR into mitochondria, and was shown to correlate with sensitivity of thymocytes to GC-mediated apoptosis [12,13].

Unfortunately, chronic administration of GCs is associated with deleterious side effects, including diabetes, osteoporosis, muscle wasting and mood swings, and severely hampers the quality of life of patients [14,15]. Prolonged GC treatment also leads to the emergence of GC resistance, a condition in which the therapeutic effects are halted, but the side effects are largely maintained [3,16,17], and of which the underlying mechanisms are diverse and incompletely resolved [8,15,18–21].

In this context, a series of selective GR agonists and modulators (SEGRAMs) were developed, with a more specific action radius compared to classics GCs and which favor transrepression over transactivation [15,22]. The development of SEGRAMs was instigated by the idea that most GC-related side effects arise from transactivation and that the anti-inflammatory properties are largely coupled to transrepression [23]. However, this dissociation needs to be nuanced, as transactivation of anti-inflammatory genes, e.g. *SPHK1*, was shown to be important in combatting acute lung inflammation [24], and since GC-related osteoporosis not solely results from GR transactivation but also from transrepression mechanisms [15,25].

Compound A (CpdA), a stable analogue of the plant-derived aziridine precursor found in a Namibian shrub [26], is the prototypical example of a SEGRM with a completely dissociated profile, that only supports transrepression and does not enhance transcription of GRE-driven genes [27–29]. CpdA has been successfully applied *in vivo* improving the disease parameters in mouse models of different inflammatory disorders [27,30–35]. CpdA was also shown to have anti-cancer properties, as it induced apoptosis in prostate, bladder, leukemia and multiple myeloma cells [4,28,36–39]. Recently, our group demonstrated in A549 cells that CpdA could modulate Dex-bound GR by enhancing both anti-inflammatory GRE-driven gene expression and the suppression of pro-inflammatory gene expression [29]. Moreover, in contrast to classic GCs, prolonged CpdA treatment does not induce homologous downregulation of GR in human synovial fibroblasts [16] and human leukemia and lymphoma cell lines [39].

As GR-mediated transrepression is an important mechanism in GC-induced cell killing, i.e. by inhibiting the expression of several anti-apoptotic genes [5], we investigate whether combining Dex with transrepression-favoring CpdA can enhance GC-mediated apoptosis. We also explore whether CpdA can protect GR protein levels from Dex-instigated homologous downregulation, which could, in extension, prolong GC responsiveness of lymphoid malignant cells.

## Materials and methods

### Cell lines and reagents

MM1.S, MM1.R, CEM-C7-14 and CEM-C1-15 cells were cultured in RPMI1640 GlutaMAX (Gibco, life technologies), supplemented with 10% fetal calf serum (Greiner bio-one), 100U/ml penicillin and 0.1mg/ml streptomycin (Gibco, life technologies), and grown in a 5% CO_2_ incubator at 37°C. MM1.S, MM1.R were purchased from ATCC, CEM-C7-14 (C7-14) and CEM-C1-15 (C1-15) cells were a kind gift from Prof. Brad E. Thompson (University of Texas Medical branch). All experiments were performed using charcoal-stripped serum (Gibco, life technologies). All cell lines were regularly tested for mycoplasma contamination and were negative.

Dexamethasone (Dex) was purchased from Sigma Aldrich, dissolved in ethanol (EtOH) and stored at -20°C. Compound A (CpdA) was synthesized as described by Louw *et al*.[26], dissolved in EtOH, flushed with an inert gas (N_2_-vapours), protected from light and was stored at -80°C. Recombinant murine TNFα, obtained from the VIB protein service facility, was dissolved in cell culture medium and used at a final concentration of 2000IU/ml. The total solvent concentration in all experiments was kept equal in each condition.

### RT-qPCR

Total RNA was isolated using an RNeasy mini kit (Qiagen), according to the manufacturer’s instructions. The resulting RNA concentration was measured using a Biodrop (Isogen). Reverse transcription (RT) was performed using an iScript cDNA synthesis kit (Bio-Rad). The resulting cDNA was used as a template for quantitative PCR (qPCR) reactions using the Lightcycler 480 SYBR Green I Master mix (Roche diagnostics), following the manufacturer’s protocol. The qPCR reaction protocol includes: a) activation enzyme and initial denaturation, 5’ at 95°C; b) 40 cycles of denaturation 15” at 95°C, hybridization and elongation 45” at 60°C, and was performed on a Lightcycler 480 system (384-well plate format, Roche diagnostics). The primer sequences are available in S1 Table. Each condition was performed in triplicate and the resulting Cq values were analyzed using qBasePlus (Biogazelle) and normalized to the reference genes: *SDHA*, *RPL13A* and *YWHAZ* [40,41]. Statistical analyses were performed on log transformed data.

### Protein lysates and Western blotting (WB)

Protein lysates were prepared using Totex lysis buffer (Hepes/KOH pH = 7.9 20mM, NaCl 350mM, glycerol 20%, NP-40 1%, MgCl_2_ 1mM, EDTA 0.5mM, EGTA 0.1mM) freshly supplemented with Halt protease and phosphatase inhibitor cocktail, EDTA-free (Thermo scientific). The samples’ protein concentration was measured via the Lowry method using the DC protein assay (Bio-Rad). 25μg (or less) of total protein was denatured, loaded on a SDS-PAGE gel, and blotted on a nitrocellulose membrane (Bio-Rad), followed by standard antibody probing procedures (Santa Cruz Biotechnology).

As primary antibodies, we used anti-GR (H300) (cat nr: sc-8992, Santa Cruz Biotechnology), anti-PARP (cat nr: 556494, BD Biosciences), anti-cleaved caspase 3 (cat nr: 9664, Cell Signaling), anti-GAPDH (cat nr: ab9485, Abcam), anti-GAPDH (cat nr: G8795, Sigma), and anti-tubulin (cat nr: T5168, Sigma). The latter three antibodies were used as loading controls. As secondary antibodies, we used species-specific HRP-conjugated antibodies (cat nr: NA931, NA934, GE-Healthcare). To visualize results, Pierce ECL (Thermo Fisher Scientific), Westernbright Quantum or Sirus (Isogen) served as chemiluminescent substrates and signals were developed using X-Ray films (GE healthcare) or imaged on a ProXima 2850 imaging system (Isogen).

### Nucleus-cytoplasm fractionation

After washing the cells with ice-cold PBS, the cells were lysed in hypotonic buffer (20mM HEPES pH = 7.0, 20% glycerol, 10mM NaCl, 1.5mM MgCl_2_, 0.2mM EDTA, 0.1% TritonX100, supplemented with Halt protease and phosphatase inhibitor cocktail, EDTA-free (Thermo scientific)) and centrifuged for 10min at 4°C and 80rcf, separating the cytoplasmic from the nuclear fraction. Next, the pelleted nuclei were resuspended in hypertonic buffer (hypotonic buffer supplemented with 500mM NaCl) to disrupt the nuclear membrane and rotated for 30min at 4°C, followed by centrifugation for 5min at 4°C and 21130rcf. From each fraction, the protein concentration was determined and maximally 10μg of total protein was further processed for WB analysis.

### MTT assay

After 72h of treatment (see figure legends), MTT (3-(4,5-dimethylthiazolyl-2)-2,5-diphenyl-2H-tetrazolium bromide, Sigma-Aldrich, 5mg MTT/ml PBS) solution (40μl/well) was added to the cells and incubated for 3-4h in a 37°C incubator protected from light, giving rise to insoluble (purple) formazan crystals in living cells. Next, SDS-HCl (10% SDS, 0.01M HCl) was added to solubilize the crystals (100μl/well) and the plate was incubated overnight at room temperature, protected from light and without a plate lid to allow equal evaporation. Absorbance (570nm with 650nm background correction) was measured using a Spectramax Paradigm spectrophotometer (Beckman Coulter) with SoftMaxPro 6.1 software.

### CellTiter-Glo and Caspase-Glo 3/7 assays

After 72h of treatment (see figure legends), cells were subjected to a CellTiter-Glo cell viability or a Caspase-Glo 3/7 assay (Promega). The CellTiter-Glo or Caspase-Glo 3/7 reagent was reconstituted by adding the CellTiter-Glo or Caspase_Glo 3/7 substrate to the CellTiter-Glo or Caspase_Glo 3/7 buffer, respectively, and was equilibrated at room temperature. A volume of CellTiter-Glo or Caspase_Glo 3/7 reagent equal to the volume in the well was added. The contents were mixed on an orbital shaker to induce cell lysis and the plate was incubated for 10’ (CellTiter-Glo) or 1h (Caspase_Glo 3/7) to stabilize the luminescent signal. Luminescence was recorded using a Spectramax Paradigm spectrophotometer (Beckman Coulter) with SoftMaxPro 6.1 software.

### Cell proliferation assay

At the indicated time points, cells were stained with trypan blue (1:1 ratio with cells) and counted using the Countess automated cell counter (Invitrogen).

### Statistical analyses

Results are presented as scatter, dot or bar plots, in which the mean +/- standard error of the mean (SEM) are depicted. When scatter dot plots are used, the open circles (o) represent the mean of the individual biological replicates. Statistical analyses were performed using GraphPad Prism 7 software. When the means of 2 groups were compared, a two-tailed unpaired t-test was used. When the means of 2 variables (e.g. induction and concentration) of more than 2 groups were compared, a two-way ANOVA with Tukey’s or Sidak’s multiple comparisons post-test was used. Results were designated significant when the *P*-value (*P*) < 0.05: * = *P* < 0.05, ** = *P* < 0.01, *** = *P* < 0.001, **** = *P* < 0.0001, ns = non-significant.

## Results

### CpdA does not support nuclear accumulation of GR yet induces HSP70 gene expression

**Fig 1:**
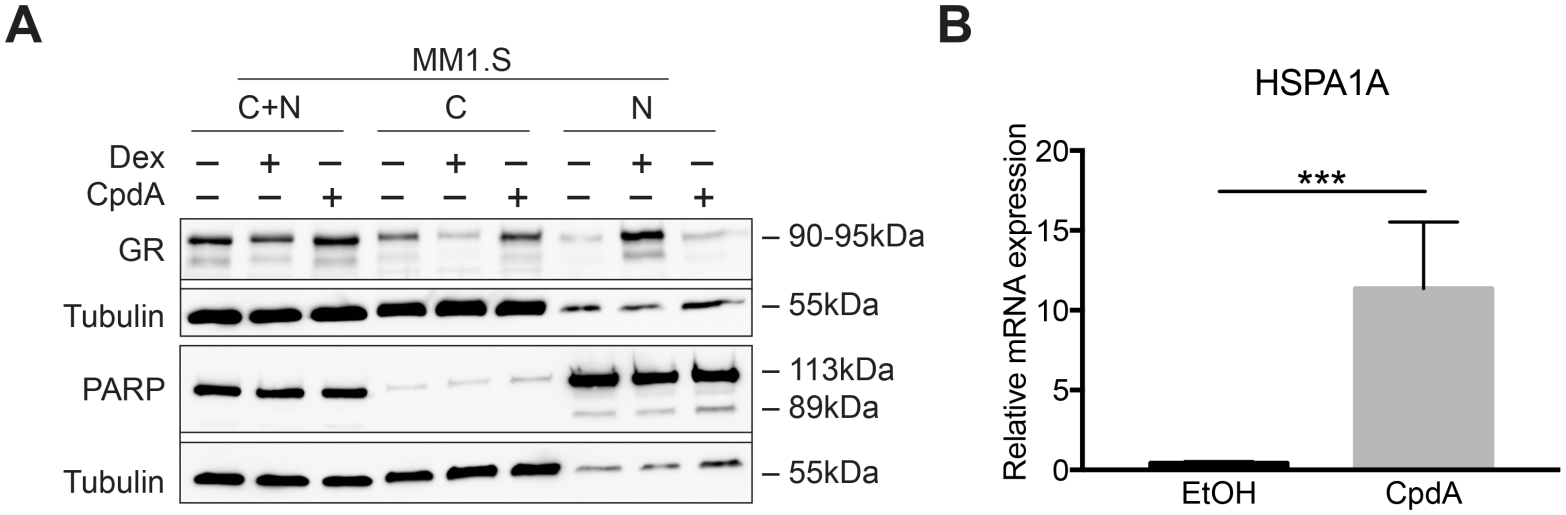
CpdA does not support GR nuclear accumulation yet induces HSP70 gene expression in MM1.S cells. (A) MM1.S cells were treated for 2h with Dex (10^-6^M) or CpdA (5.10^-6^M) and Nucleus (N)–Cytoplasm (C) fractionation was performed. Protein lysates were prepared and WB analysis was performed, detecting the protein levels of GR (90-95kDa) and PARP (89 and 113 kDa, N fraction control), with tubulin (55kDa, C fraction control) serving as loading control. WB results are representative of 2 independent experiments. (B) MM1.S cells were treated for 6h with CpdA (10^-5^M). RNA was isolated and subjected to RT-QPCR, detecting the mRNA levels of *HSPA1A* with *SDHA*, *YWHAZ* and *RPL13A* serving as reference genes. The bar plot represents the mean +/- SEM of 5 biological replicates. A two-tailed unpaired t-test was performed on log transformed data using GraphPad Prism 7. *** = *P* < 0.001.

CpdA was shown to induce nuclear translocation of GR in different cell models, albeit to varying degrees [27,28,42]. Therefore, we first assayed nuclear accumulation of GR upon CpdA treatment in GC-sensitive MM1.S cells. We monitored the degree of cellular fractionation by determining PARP and tubulin levels, as controls for nuclear and cytoplasmic fractions, respectively (Fig 1A). As a positive control, Dex is used as it strongly induces nuclear translocation of GR [43]. In contrast, Fig 1A shows that CpdA does not support a marked nuclear accumulation of GR.

Recently, Beck *et al*. reported that CpdA induces Hsp70 gene expression in A549 cells in a GR-dependent manner [44]. Hence, as a control for the activity of CpdA, we monitored the expression levels of *HSPA1A*, one of the genes coding for Hsp70 [45], in MM1.S after 6h treatment with CpdA. As shown in Fig 1B, CpdA strongly induces *HSPA1A* expression compared to solvent control.

### Adding CpdA to Dex treatment does not substantially enhance GC-mediated cell killing

**Fig 2:**
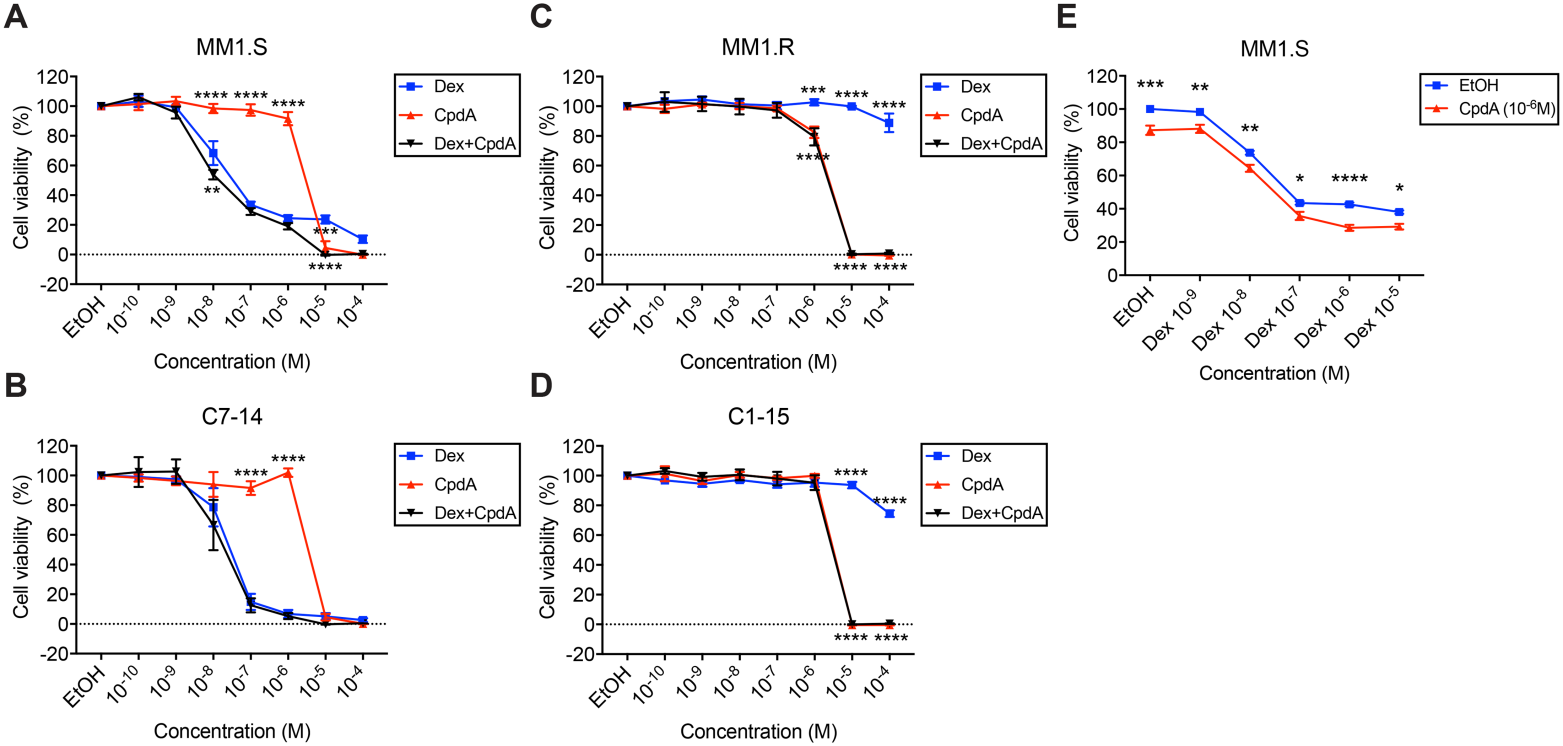
Adding CpdA to Dex treatment does not substantially enhance GC-induced cell killing in GC-sensitive and GC-resistant MM and ALL cells. (A) MM1.S and (B) C7-14 cells, (C) MM1.R and (D) C1-15 cells were treated for 72h with a concentration range (10^-4^M-10^-10^M) of Dex, CpdA or Dex/CpdA combinations (equimolar concentrations). Cell viability was determined using MTT assays. (E) MM1.S cells were treated for 72h with fixed concentration CpdA (10^-6^M) and/or with a concentration range (10^-5^M-10^-9^M) of Dex. Cell viability was determined using a CellTiter-Glo assay. The cell viability of the solvent control (EtOH) was set at 100% and all other cell viabilities were normalized accordingly. The scatter plots represent the mean +/- SEM of 4 independent experiments. Statistical analysis was performed using GraphPad Prism 7, using a two-way ANOVA with Tukey’s (A-D) or Sidak’s (E) multiple comparison post-test, comparing Dex vs. CpdA or Dex vs. Dex/CpdA per concentration. Only significant differences are displayed: * = *P* < 0.05, ** = *P* < 0.01, *** = *P* < 0.001, **** = *P* < 0.0001.

Next, we determined GC responsiveness of the GC-sensitive MM1.S (MM) and C7-14 (ALL) cells and the GC-resistant MM1.R (MM) and C1-15 cells (ALL) [46,47]. We treated these cells for 72h with a concentration range (10^-4^M-10^-10^M) of Dex, CpdA or the combination hereof and measured cell viability using MTT assays. Fig 2A and 2B show that the cell viability of GC-sensitive MM1.S and C7-14 cells decreases with Dex treatments in a concentration-responsive manner. As expected, GC-resistant MM1.R and C1-15 do not respond to Dex treatment (Fig 2C and 2D). In addition, CpdA alone does not decrease the cell viability of GC-sensitive MM1.S and C7-14 cells (Fig 2A and 2B), except slightly (10–20%) at 10^-6^M in MM1.S and MM1.R cells and in all cell lines at the higher (10^-4^M and 10^-5^M) concentrations. This mild effect of 10^-6^M CpdA on cell viability seems GR-independent as it occurred in both GR-positive MM1.S cells and in GR-negative MM1.R cells. Also, cell killing mediated by the highest CpdA concentrations (10^-4^M, 10^-5^M) is likely due to GR-independent effects, as it is also observed in the GC-resistant, GR-positive C1-15 cells as well as in the GR-negative MM1.R cells (Fig 2C and 2D). The combination of Dex and CpdA shows a similar concentration-responsive reduction of MM1.S and C7-14 cell viabilities as Dex treatment alone. Alternatively, cell viability was determined using CellTiter-Glo assays and a similar concentration range (10^-5^M-10^-9^M) of Dex, CpdA and the combination hereof. In accordance with the MTT results, addition of CpdA to Dex treatment does not further decrease the cell viability of GC-sensitive MM and ALL cells (S1 Fig).

In addition, this experiment was repeated with a fixed (lower) concentration of CpdA (10^-6^M) and varying concentrations (10^-5^M-10^-9^M) of Dex. Fig 2E shows that combined Dex and CpdA treatments leads to a minor reduction (on average 10%) of MM1.S cell viability at each concentration, yet, overall no statistical interaction can be shown between Dex and CpdA.

### Dex and CpdA combination neither augments GC-induced apoptosis nor increases cell proliferation inhibition

**Fig 3:**
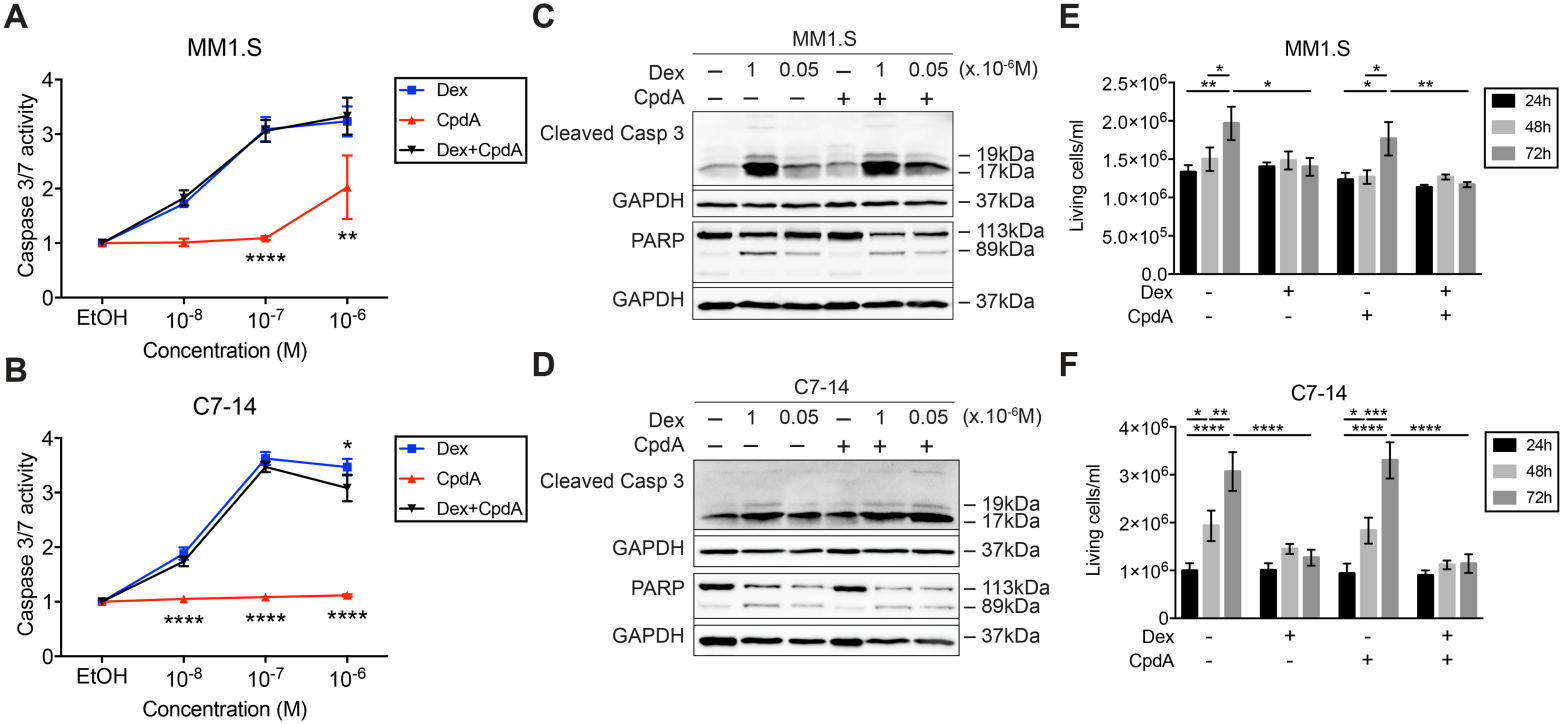
Adding CpdA to Dex treatment neither increases GC-induced apoptosis nor additionally inhibits cell proliferation of GC-sensitive MM and ALL cells. (A) MM1.S and (B) C7-14 cells were treated for 72h with a concentration range (10^-6^M-10^-8^M) of Dex, CpdA or Dex/CpdA combinations (equimolar concentrations). Caspase activity was determined using Caspase-Glo 3/7 assays. The caspase 3/7 activity of the solvent control (EtOH) was set at 1 and all other values were normalized accordingly. The scatter plots represent the mean +/- SEM of 4 (A) or 3 (B) independent experiments. Statistical analysis was performed using GraphPad Prism 7, using a two-way ANOVA with Tukey’s multiple comparison post-test, comparing Dex vs. CpdA or vs. Dex/CpdA per concentration. Only significant differences are displayed: ** = *P* < 0.01, **** = *P* < 0.0001. (C) MM1.S and (D) C7-14 cells were treated for 72h with Dex (10^-6^M or 5.10^-8^M), CpdA (5.10^-6^M) or Dex/CpdA combination. Protein lysates were prepared and subjected to WB analysis, assaying protein levels of cleaved caspase 3 (17 and 19 kDa), PARP (89 and 113kDa), with GAPDH (37kDa) serving as loading control. WB results are representative of 2 independent experiments. (E) MM1.S and (F) C7-14 cells were treated for 24h, 48h or 72h with Dex (10^-6^M), CpdA (5.10^-6^M) or Dex/CpdA combination. At the corresponding time points, the cells were stained with trypan blue and counted using the Countess automated cell counter. The bar plots represent the mean +/- SEM of 3 independent experiments. Statistical analysis was performed using GraphPad Prism 7, using a two-way ANOVA with Tukey’s multiple comparison post-test. Only significant differences are displayed: * = *P* < 0.05, ** = *P* < 0.01, *** = *P* < 0.001, **** = *P* < 0.0001.

We further zoomed in on GC-mediated apoptosis and evaluated whether 72h treatment with a limited concentration range (10^-6^M-10^-8^M) of Dex, CpdA or the combination of both could increase caspase 3/7 activity of GC-sensitive MM1.S and C7-14 cells. Fig 3A and 3B shows that Dex treatment elevates the caspase 3/7 activity in MM1.S and C7-14 cells with increasing concentrations. Yet, maximal caspase 3/7 activity in C7-14 cells is reached at lower concentration Dex (10^-7^M) compared to MM1.S cells (10^-6^M). CpdA treatment does not augment caspase 3/7 activity at any concentration in C7-14 cells, while in MM1.S cells this activity is slightly enhanced at 10^-6^M CpdA (Fig 3A and 3B). Equimolar Dex and CpdA combination also do not augment caspase 3/7 activity compared to Dex treatment in MM1.S or C7-14 cells. In addition, we repeated this experiment with a fixed concentration of CpdA (10^-5^M) and varying concentrations (10^-6^M-10^-8^M) of Dex. S2 Fig shows that 10^-5^M CpdA induces no caspase 3/7 activity at 72h and is probably even cell-toxic, as the caspase 3/7 activity drops markedly under that of the EtOH control in each condition where CpdA is added, an observation in line with Fig 2. In contrast, at 24h and 48h of treatment, 10^-5^M CpdA induces cleavage of PARP and caspase 3 (apoptosis hallmarks) in MM1.S cells, yet, not at 10^-6^M and 10^-7^M CpdA. Also, at 48h, 10^-5^M CpdA becomes cytotoxic for the cells as evidenced by the decreased GAPDH levels.

Protein levels of other apoptosis hallmarks, such as PARP cleavage and cleaved-caspase 3, were also assayed following 72h treatment with Dex (10^-6^M or 5.10^-8^M), CpdA (5.10^-6^M) or a combination thereof. CpdA treatment alone does not change the cleaved-caspase 3 levels, and PARP cleavage is not induced in both MM1.S and C7-14 cells (Fig 3C and 3D). Cleaved-caspase 3 levels are the highest and PARP cleavage is the strongest with 10^-6^M Dex treatment and added CpdA does not change these levels (Fig 3C and 3D), possibly because a plateau is reached. In line with this hypothesis, combining 5.10^-8^M Dex with CpdA increases the levels of cleaved-caspase 3 compared to 5.10^-8^M Dex alone in both cell lines, yet, this is not the case for PARP cleavage (Fig 3C and 3D).

In addition, cell proliferation was assayed by treating MM1.S and C7-14 cells for 72h with Dex (10^-6^M), CpdA (5.10^-6^M) or both combined. As expected, the number of living cells increases in the control condition in function of time, indicative of proliferating cells (Fig 3E and 3F). In contrast, Dex treatment blocks proliferation of the cells, which is most pronounced at 72h in C7-14 cells. CpdA does not affect cell proliferation as the number of living cells/ml is comparable to the control condition. Consistently, Fig 3E and 3F show that the combination of Dex and CpdA largely reflects the number of living cells/ml of Dex treatment alone.

### GR protein levels are not sustained by combining Dex and CpdA

**Fig 4:**
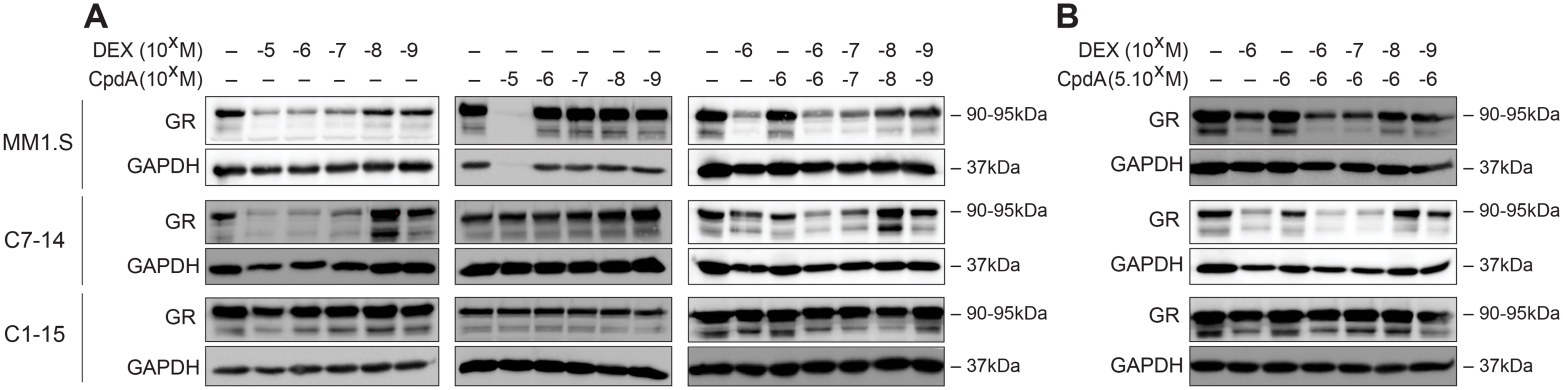
GR protein levels are not preserved by combining Dex with CpdA in GC-sensitive MM and ALL cells, or in GC-resistant ALL cells. MM1.S, C7-14 and C1-15 cells were treated for 72h with (A) a concentration range (10^-5^M-10^-9^M) of Dex, CpdA or Dex/CpdA combination or (B) a concentration range of Dex (10^-6^M-10^-9^M), CpdA (5.10^-6^M) or Dex/CpdA combination. Protein lysates were prepared and WB analysis was performed, detecting the protein levels of GR (90-95kDa), with GAPDH (37kDa) serving as a loading control. WB results are representative of 3 independent experiments.

CpdA was previously shown to protect GR from homologous downregulation in various cells [16,39]. Therefore, we wondered whether the addition of CpdA to Dex treatment might sustain GR protein levels in MM1.S, C7-14 and C1-15 cells. To this end, cells were treated for 72h with a concentration range (10^-5^M-10^-9^M) of Dex, CpdA or a combination hereof (Fig 4A), and GR protein levels were determined. MM1.R cells are not taken along as they are GR-negative (S3A Fig). GR levels in GC-resistant C1-15 cells remain largely unaltered, regardless of any treatment (Fig 4A and 4B). In MM1.S and C7-14 cells, GR protein levels decrease by Dex treatment in a concentration-responsive manner, except in C7-14 cells at 10^-8^M Dex, where GR levels even increase compared to control. Although this 10^-8^M Dex-effect is already present after 24h, the increase in GR levels is most pronounced after 72h (S3B Fig). CpdA treatment alone does not affect GR protein levels, but again kills cells at 10^-5^M in MM1.S cells, as evident from the lacking GAPDH levels (Fig 4A). Equimolar concentrations of CpdA and Dex is unable to rescue GR from degradation in MM1.S and C7-14 cells, and GR levels largely resemble those of Dex treatment alone.

To exclude that the latter is due to the use of equimolar concentrations, we treated the cells with a fixed CpdA concentration on top of varying Dex concentrations (10^-6^M-10^-9^M). Fig 4B shows that using this treatment scheme, GR protein levels are also not protected from degradation and are strongly reduced in a concentration-dependent manner compared to control.

### CpdA does not alter Dex-mediated transactivation of GRE-driven genes

**Fig 5:**
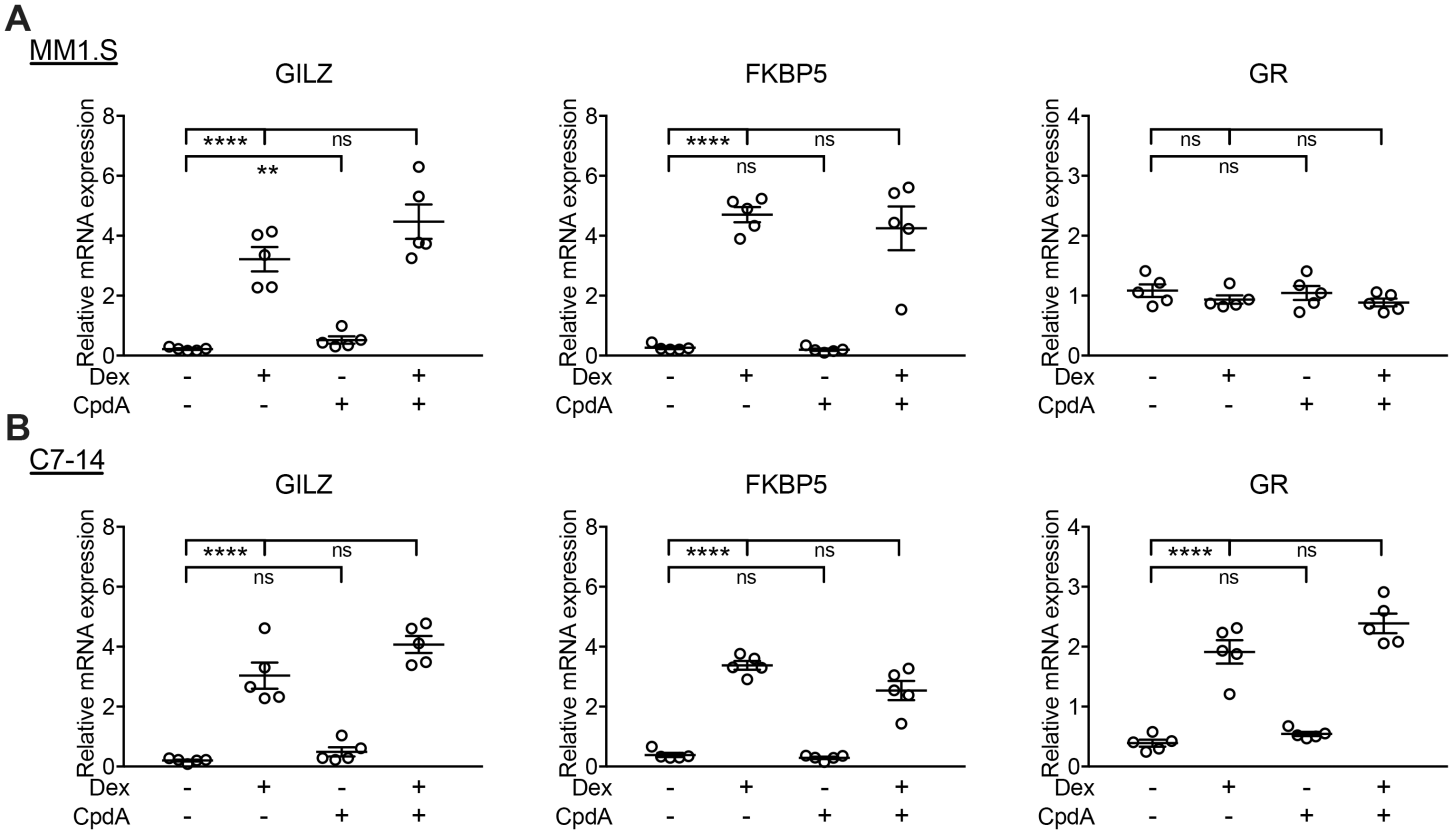
CpdA does not modulate Dex-induced GR transactivation of GRE-driven genes in GC-sensitive MM and ALL cells. (A) MM1.S and (B) C7-14 cells were treated for 6h with Dex (10^-6^M), CpdA (10^-5^M) or Dex/CpdA combination. RNA was isolated and subjected to RT-QPCR, detecting the mRNA levels of *GILZ*, *FKBP5* and *GR* and with *SDHA*, *YWHAZ* and *RPL13A* serving as reference genes. The dot plots represent the mean +/- SEM of 5 biological replicates, with the open circles (o) representing the mean of each biological experiment. A two-way ANOVA with Tukey’s multiple comparison post-test was performed on log transformed data using GraphPad Prism 7. ** = *P* < 0.01, **** = *P* < 0.0001, ns = non-significant.

Our group recently reported that CpdA can modulate Dex-instigated gene expression [29]. To test whether this also occurs in lymphoid cell lines, cells were treated for 6h with Dex, CpdA or the combination hereof, followed by QPCR analyses of GR and of its target genes *GILZ* and *FKBP5*. Both *GILZ* and *FKBP5* are strongly induced upon Dex treatment in MM1.S and C7-14 cells (Fig 5) but also in C1-15 cells (S4A Fig), the latter regardless of the cells’ resistance towards GC-mediated apoptosis. *GR* mRNA levels are unaffected by 6h Dex treatment in MM1.S cells, but are upregulated in C7-14 and C1-15 cells (Fig 5 and S4A Fig). At large, CpdA does not support transactivation of GRE-driven genes, except for a mild increase in GILZ expression in MM1.S cells (Fig 5A). Moreover, combining Dex and CpdA does not alter the expression levels of these genes compared to Dex treatment (Fig 5), except for the *GR* mRNA levels in C1-15 cells, which are elevated compared to Dex (S4A Fig).

### CpdA does not support transrepression of pro-inflammatory genes, in presence or absence of Dex

We subsequently wondered whether CpdA supports transrepression in the lymphoid cell lines under study and whether CpdA modulates transrepression instigated by Dex-activated GR. Hence, we prestimulated the cells 1h with Dex, CpdA or a combination thereof, followed or not by 5h of a pro-inflammatory stimulus (TNFα) and assayed the mRNA levels of *A20*, *NFKBIA* (IκBα), *ICAM* and *RANTES*.

**Fig 6:**
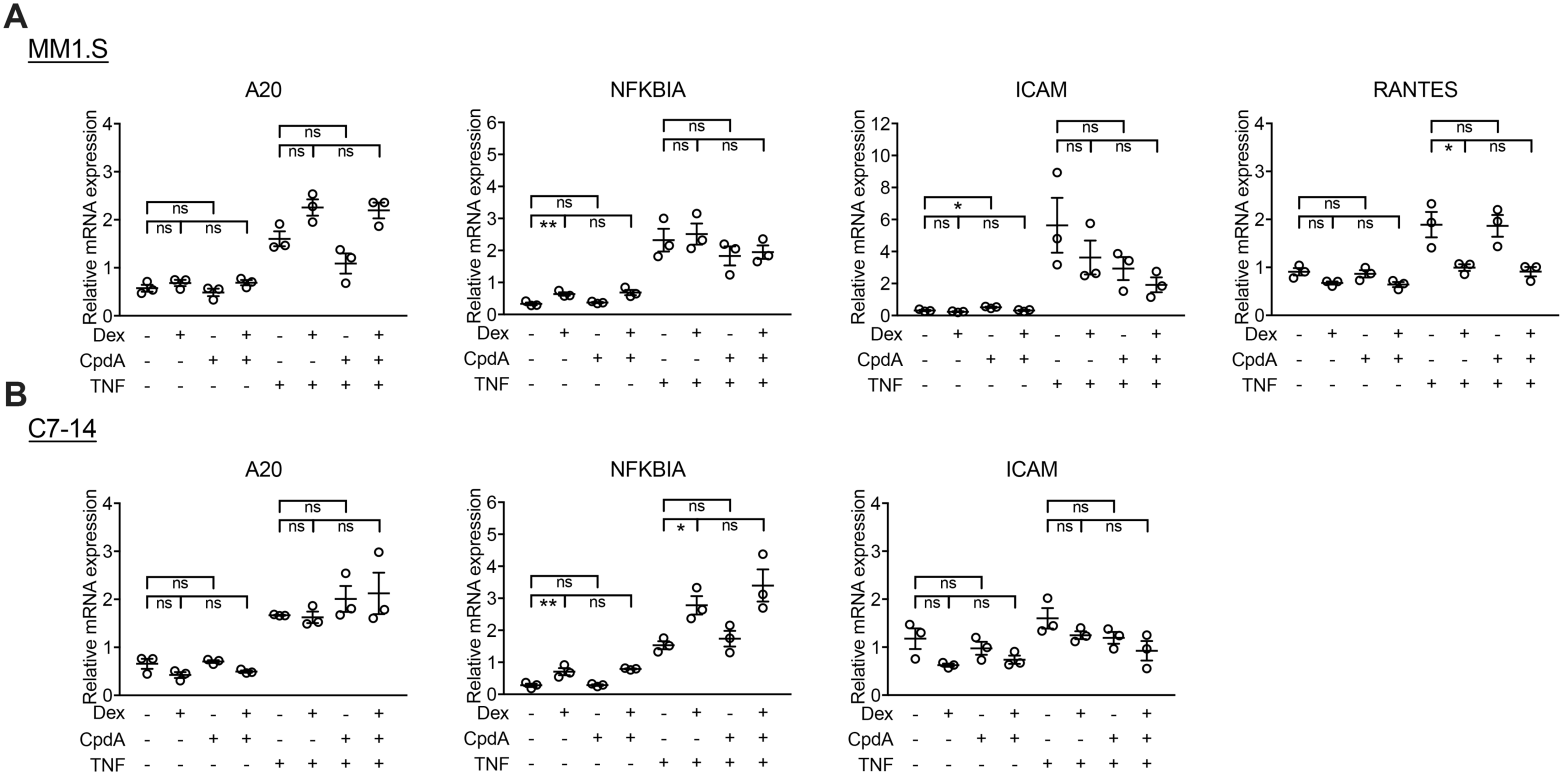
CpdA does not transrepress pro-inflammatory genes in GC-sensitive MM and ALL cells, in absence or presence of Dex. (A) MM1.S and (B) C7-14 cells were pretreated with Dex (10^-6^M), CpdA (10^-5^M) or Dex/CpdA combination for 1h, followed by or not treatment with TNFα (2000IU/ml) for another 5h. RNA was isolated and subjected to RT-QPCR, detecting the mRNA levels of *A20*, *NFKBIA*, *ICAM* and *RANTES*, with *SDHA*, *YWHAZ* and *RPL13A* serving as reference genes. The dot plots represent the mean +/- SEM of 3 biological replicates, with the open circles (o) representing the mean of each biological experiment. A two-way ANOVA with Tukey’s multiple comparison post-test was performed on log transformed data using GraphPad Prism 7. * = *P* < 0.05, ** = *P* < 0.01, ns = non-significant.

*A20* and *NFKBIA*, both key inhibitors of NF-κB, are induced upon TNFα stimulation and both have a GR- and NF-κB-binding site in their corresponding promoters [48,49] (Fig 6). Dex augments *NFKBIA* expression levels in MM1.S and C7-14 cells, and only in the latter even beyond the TNFα-induced *NFKBIA* expression (Fig 6, S4B Fig). TNFα-induced *A20* mRNA expression is not further induced by Dex. Moreover, CpdA does not significantly decrease TNFα-stimulated *A20* or *NFKBIA* mRNA levels in any cell line. Also, the combination of Dex and CpdA does not differentially affect the TNFα-induced expression of *A20* and *NFKBIA* compared to Dex treatment (Fig 6, S4B Fig).

The pro-inflammatory genes *ICAM* and *RANTES* are upregulated by TNFα treatment, while Dex only mildly reduces this stimulation for *RANTES* in MM1.S cells (Fig 6A). Remarkably, in MM1.S cells CpdA stimulates *ICAM* expression in the absence of TNF, albeit mildly. Nevertheless, in all cell lines, CpdA is unable to significantly reduce TNFα-stimulated expression of *ICAM* and *RANTES* and also the combination of Dex and CpdA does not result in further inhibition of TNFα-induced pro-inflammatory gene expression compared to Dex (Fig 6, S4B Fig).

## Discussion

The potential of SEGRAMs to reduce the number and intensity of GC-coupled side effects with improved therapeutic efficacy and prolonged responsiveness [3,15], is an attractive route to explore in lymphoid malignancies. Therefore, we investigated whether the combination of classic GCs with transrepression-favoring SEGRAMs could promote GC-induced apoptosis and/or postpone GC resistance by protecting GR from degradation.

CpdA did not substantially induce GRE-driven gene expression in MM1.S, C7-14 or C1-15 cells, in accordance with previous reports on other cell types [27,28,30,50]. The lack of CpdA to induce GR dimerization was proposed to underlie its dissociated behavior [30,42] and was also linked to decreased nuclear import and increased nuclear export [51]. In line herewith, CpdA indeed did not lead to a marked GR nuclear enrichment in MM1.S cells. Varying degrees of CpdA-instigated GR nuclear translocation were also reported in LNCaP-GR prostate cancer cells [28], CT5.3hTERT cancer-associated fibroblasts [52] and A549 cells [29], and thus seems a cell type-dependent phenomenon. In addition, CpdA’s dissociated behavior could arise from a different conformational change in GR, leading to altered coregulator recruitment and slightly altered target gene preferences [27,29,53,54]. Consistent herewith, CpdA increased *GILZ* and *ICAM* expression in MM1.S and C7-14 cells, respectively. Alternatively, CpdA is able to GR-independently upregulate *DUSP1* in airway smooth muscle cells as part of a mechanism by which CpdA blocks production of GC-resistant chemokines [55]. Similarly, Desmet *et al*. observed a CpdA-mediated upregulation of *DUSP1* in intestinal epithelial cells and to a lesser extent in A549 cells [29]. In addition, *HSPA1A* mRNA was upregulated by CpdA in MM1.S cells, which was also reported for A549 cells, albeit as a GR-dependent phenomenon [44]. The fact that DNA itself can act as a sequence-specific allosteric regulator of GR [56], might be an additional contributor as to why certain GR ligands discriminate between GR transactivation of particular genes. Principally, CpdA could not modulate Dex-induced transactivation, except for an increased GR expression in C1-15 cells, as compared to Dex, again suggesting cell type- and gene-specific modulation.

TNF-induced pro-inflammatory gene expression was only modestly reduced by Dex in MM1.S cells and additional CpdA did not alter these Dex effects. Also CpdA alone failed to inhibit TNF-induced pro-inflammatory gene expression. This aberrant transrepression profile on top of a lack in transactivation may underpin why adding CpdA to Dex treatment largely failed to enhance GC-mediated apoptosis and to additionally inhibit cell proliferation of these lymphoid cell lines. The actual mechanisms underlying GC-induced apoptosis in lymphoid cells are not completely elucidated [4,57]. For instance, there is no consensus whether either transactivation of pro-apoptotic genes (e.g. BIM, GILZ), or the transrepression of pro-inflammatory genes (e.g. IL-6), anti-apoptotic genes (e.g. Bcl-XL) and cell cycle promoting genes (e.g. cyclin D1), is the most crucial mechanism governing GC-induced apoptosis [8,18,19,21,58,59]. It is most likely that both mechanisms contribute to GC-mediated cell death and that also non-genomic mechanisms are important [12]. Anyhow, we found that GC-induced killing of lymphoid cells was only slightly strengthened by adding (10^-6^M) CpdA and given its dissociated profile, this suggests that transrepression alone is most likely not sufficient to provoke GC-mediated apoptosis. In addition, CpdA alone has two different effects depending on the concentration that is used: 10^-4^M-10^-5^M CpdA (high concentration) is cytotoxic, while 10^-6^M CpdA and lower concentrations are not. These results differ from the studies of Lesovaya and coworkers, showing CpdA induced PARP cleavage and upregulated Bim and p53 expression in leukemia cells [38], with nanomolar amounts of CpdA able to reduce the cell growth of MM1.S cells [39]. However, in the same study CpdA also reduced the cell growth of GR-negative MM1.R cells at 10^-7^M [39], indicating that the observed results might have a GR-independent component.

In contrast, we only observed massive cell death with CpdA at very high concentrations (10^-4^M-10^-5^M), which was independent of the cells’ GR status and responsiveness to GCs. This agrees with Wüst and colleagues, who showed that various cell types (lymphocytes, fibroblasts, neuronal cells) can undergo massive GR-independent cell death with high dose CpdA [31]. This is ascribed to CpdA’s instability and thus its cyclization into an aziridine intermediate, which is known to have alkylating properties. This process especially occurs in buffers (e.g. PBS at higher pH) and after longer incubation periods [31]. However, this does not mean that in another study CpdA’s effects are also per se GR-independent, as this most definitely also depends on the cell context (e.g. inflammation vs. cancer) and cell type.

Prolonged treatment with CpdA was shown to protect GR from homologous downregulation in fibroblast-like synoviocytes, isolated from rheumatoid arthritis patients. Treatment for 24h with CpdA sustained GR protein levels, while Dex already downregulated GR levels after 6h [16]. CpdA treatment of CEM and NCEB cells for 24h also preserved GR levels [39]. In contrast, here, addition of CpdA to Dex treatment was unable to prevent homologous downregulation of GR in lymphoid cell lines. The proposed combination strategy is thus unable to prolong GC responsiveness in lymphoid cell lines via sustained and protected GR protein levels.

Besides in lymphoid malignancies, CpdA was also described to have anti-cancer properties in solid cancers [4]. In prostate cancer, CpdA was shown to act as a combined AR antagonist and GR agonist, resulting in the inhibition of prostate tumor growth and the induction of apoptosis *in vitro* [28]. The latter effect was even more pronounced when CpdA was combined with Bortezomib, as this proteasome inhibitor results in GR accumulation [36]. Also in bladder cancer, CpdA was reported to inhibit cell proliferation and induce cell cycle arrest and apoptosis in GR+/AR+ cells, and to reduce tumor growth more strongly than Dex in a xenograft model [37]. In addition, Chen and coworkers demonstrated that in triple negative breast cancer (TNBC), where GCs are given as adjuvant, CpdA regulates only a small number of genes that are not involved in carcinogenesis. This is in sharp contrast to Dex, which regulates a large set of genes that are associated with TNBC progression and drug resistance [60]. The latter exemplifies the potential for SEGRAMs in solid tumors.

Taken together, we favor CpdA’s classification as a selective GR modulator (not ligand), which refers to its dissociated behavior, meaning that CpdA supports GR transrepression but not GR transactivation. Yet, this terminology does not exclude alternative action modes of CpdA such as non-genomic mechanisms, or targeting of other nuclear receptors or transcription factors. Finally, our study demonstrates that dissociated, transrepression-favoring compounds, such as CpdA, do not seem a valid therapeutic strategy in the treatment of lymphoid malignancies, but can hold promise for the treatment of solid cancers and inflammation.

## Supporting information

S1 Table RT-qPCR primer sequences. (DOCX)

S1 Fig Effect of Dex, CpdA and Dex/CpdA combination treatment on the cell viability of GC-sensitive MM and ALL cells. (A) MM1.S (MM) and (B) C7-14 (ALL) cells were treated for 72h with a concentration range (10^-5^M-10^-9^M) of Dex, CpdA or Dex/CpdA combination (equimolar concentrations). The cell viability was determined using CellTiter-Glo assays. The cell viability of the solvent control (EtOH) was set at 100% and all other cell viabilities were normalized accordingly. The scatter plots represent the mean +/- SEM of 3 independent experiments. Statistical analysis was performed using GraphPad Prism 7, using a two-way ANOVA with Tukey’s multiple comparison post-test, comparing Dex vs. CpdA or vs. Dex/CpdA per concentration. Only significant differences are displayed: * = *P* < 0.05, ** = *P* < 0.01, **** = *P* < 0.0001.
(TIF)

S2 Fig Effect of Dex, CpdA and Dex/CpdA combination treatment on GC-induced apoptosis of GC-sensitive MM and ALL cells. (A) MM1.S and (B) C7-14 cells were treated for 72h with a Dex concentration range (10^-6^M-10^-8^M), CpdA (10μM) or Dex/CpdA combination (fixed CpdA concentration). The caspase activity was determined using Caspase-Glo 3/7 assays. The caspase 3/7 activity of the solvent control (EtOH) was set at 1 and all other values were normalized accordingly. The scatter plots represent the mean +/- SEM of 3 independent experiments. Statistical analysis was performed using GraphPad Prism 7, using a two-way ANOVA with Sidak’s multiple comparison post-test, comparing Dex vs. Dex/CpdA per concentration. Only significant differences are displayed: ** = *P* < 0.01. (C) MM1.S cells were treated for 24h or 48h with solvent, 10^-6^M Dex or a limited CpdA concentration range (10^-5^M-10^-7^M). Protein lysates were subjected to WB analysis, determining the protein levels of PARP (89 and 113kDa) and cleaved-caspase 3 (17-19kDa), with GAPDH (37kDa) serving as loading control. Results are representative of 3 independent experiments.
(TIF)

S3 Fig Absence of GR in MM1.R cells and Dex concentration range of C7-14 cells in function of time. (A) MM1.R cells were treated for 72h with a Dex concentration range (10^-4^M-10^-10^M). (B) C7-14 cells were treated for 24h, 48h or 72h with a Dex concentration range (10^-7^M-10^-9^M). (A-B) Protein lysates were prepared and WB analysis was performed, detecting the protein levels of GR (90-95kDa), with GAPDH (37kDa) serving as loading control. WB results arise from 1 (A) biological experiment, or are representative of 2 (B) biological experiments.
(TIF)

S4 Fig Evaluation of transactivation and transrepression in GC-resistant ALL cells. C1-15 cells were treated for 6h with Dex (1μM), CpdA (10μM) or Dex/CpdA combination. RNA was isolated and subjected to RT-QPCR, detecting the mRNA levels of (A) *GILZ*, *FKBP5* and *GR* as a measure for transactivation and (B) *A20*, *NFKBIA*, *ICAM* and *RANTES* as a measure for transrepression. (A-B) *SDHA*, *YWHAZ* and *RPL13A* served as reference genes. The dot plots represent the mean +/- SEM of 5 (A) or 3 (B) biological replicates with the open circles (o) representing the mean of each biological experiment. A two-way ANOVA with Tukey’s multiple comparison post-test was performed on log transformed data using GraphPad Prism 7. * = *P* < 0.05, ** = *P* < 0.01, **** = *P* < 0.0001, ns = non-significant.
(TIF)

## References

[1] Tan CK, Wahli W. A trilogy of glucocorticoid receptor actions. Proceedings of the National Academy of Sciences of the United States of America. 2016;113: 1115–1117. doi: 10.1073/pnas.1524215113 26792523 10.1073/pnas.1524215113 PMC4747747

[2] Ramamoorthy S, Cidlowski JA. Corticosteroids: Mechanisms of Action in Health and Disease. Rheumatic Disease Clinics of North America. 2016;42: 15–31 26611548 10.1016/j.rdc.2015.08.002 PMC4662771

[3] De Bosscher K, Haegeman G, Elewaut D. Targeting inflammation using selective glucocorticoid receptor modulators. Current Opinion in Pharmacology. 2010;10: 497–504. doi: 10.1016/j.coph.2010.04.007 20493772 10.1016/j.coph.2010.04.007

[4] Sundahl N, Clarisse D, Bracke M, Offner F, Vanden W, Beck IM. Selective glucocorticoid receptor-activating adjuvant therapy in cancer treatments. Oncoscience. 2016;3: 22–30. doi: 10.18632/oncoscience.315 27713909 10.18632/oncoscience.315 PMC5043069

[5] Schlossmacher G, Stevens A, White A. Glucocorticoid receptor-mediated apoptosis: mechanisms of resistance in cancer cells. Journal of Endocrinology. 2011;211: 17–25. doi: 10.1530/JOE-11-0135 21602312 10.1530/JOE-11-0135

[6] Rajkumar SV. Multiple myeloma: 2016 update on diagnosis, risk-stratification, and management. American Journal of Hematology. 2016;91: 719–734. doi: 10.1002/ajh.24402 27291302 10.1002/ajh.24402 PMC5291298

[7] Inaba H, Greaves M, Mullighan CG. Acute lymphoblastic leukaemia. Lancet. 2013;381: 1943–55. doi: 10.1016/S0140-6736(12)62187-4 23523389 10.1016/S0140-6736(12)62187-4 PMC3816716

[8] Smith LK, Cidlowski JA. Glucocorticoid-induced apoptosis of healthy and malignant lymphocytes. Progress in Brain Research. 2010;182: 1–30. doi: 10.1016/S0079-6123(10)82001-1 20541659 10.1016/S0079-6123(10)82001-1 PMC4770454

[9] Ratman D, Vanden Berghe W, Dejager L, Libert C, Tavernier J, Beck IM, et al How glucocorticoid receptors modulate the activity of other transcription factors: A scope beyond tethering. Molecular and cellular endocrinology. 2013;380: 41–54. doi: 10.1016/j.mce.2012.12.014 23267834 10.1016/j.mce.2012.12.014

[10] Lim HW, Uhlenhaut NH, Rauch A, Weiner J, Hübner S, Hübner N, et al Genomic redistribution of GR monomers and dimers mediates transcriptional response to exogenous glucocorticoid in vivo. Genome Research. 2015;25: 836–844. doi: 10.1101/gr.188581.114 25957148 10.1101/gr.188581.114 PMC4448680

[11] Oh KS, Patel H, Gottschalk RA, Lee WS, Baek S, Fraser IDC, et al Anti-Inflammatory Chromatinscape Suggests Alternative Mechanisms of Glucocorticoid Receptor Action. Immunity. 2017;47: 298–309.e5. doi: 10.1016/j.immuni.2017.07.012 28801231 10.1016/j.immuni.2017.07.012 PMC5572836

[12] Kfir-Erenfeld S, Yefenof E. Non-genomic events determining the sensitivity of hemopoietic malignancies to glucocorticoid-induced apoptosis. Cancer immunology, immunotherapy. 2014;63: 37–43. doi: 10.1007/s00262-013-1477-8 24072402 10.1007/s00262-013-1477-8 PMC11028523

[13] Talaber G, Boldizsar F, Bartis D, Palinkas L, Szabo M, Berta G, et al Mitochondrial translocation of the glucocorticoid receptor in double-positive thymocytes correlates with their sensitivity to glucocorticoid-induced apoptosis. International immunology. 2009;21: 1269–1276. doi: 10.1093/intimm/dxp093 19737783 10.1093/intimm/dxp093

[14] Schacke H, Docke WD, Asadullah K. Mechanisms involved in the side effects of glucocorticoids. Pharmacology and Therapeutics. 2002;96: 23–43. doi: 10.1016/S0163-7258(02)00297-8 12441176 10.1016/s0163-7258(02)00297-8

[15] Sundahl N, Bridelance J, Libert C, De Bosscher K, Beck IM. Selective glucocorticoid receptor modulation: New directions with non-steroidal scaffolds. Pharmacology & therapeutics. 2015;152: 28–41. doi: 10.1016/j.pharmthera.2015.05.001 25958032 10.1016/j.pharmthera.2015.05.001

[16] Gossye V, Elewaut D, Van Beneden K, Dewint P, Haegeman G, De Bosscher K. A plant-derived glucocorticoid receptor modulator attenuates inflammation without provoking ligand-induced resistance. Annals of the Rheumatic diseases. 2010;69: 291–296. doi: 10.1136/ard.2008.102871 19204014 10.1136/ard.2008.102871

[17] Norman M, Hearing SD. Glucocorticoid resistance—what is known? Current Opinion in Pharmacology. 2002;2: 723–729. doi: 10.1016/S1471-4892(02)00232-1 12482737 10.1016/s1471-4892(02)00232-1

[18] Herr I, Gassler N, Friess H, Buchler MW. Regulation of differential pro- and anti-apoptotic signaling by glucocorticoids. Apoptosis. 2007;12: 271–291. doi: 10.1007/s10495-006-0624-5 17191112 10.1007/s10495-006-0624-5

[19] Gross KL, Lu NZ, Cidlowski JA. Molecular mechanisms regulating glucocorticoid sensitivity and resistance. Molecular and cellular endocrinology. 2009;300: 7–16. doi: 10.1016/j.mce.2008.10.001 19000736 10.1016/j.mce.2008.10.001 PMC2674248

[20] Yang N, Ray DW, Matthews LC. Current concepts in glucocorticoid resistance. Steroids. 2012;77: 1041–1049. doi: 10.1016/j.steroids.2012.05.007 22728894 10.1016/j.steroids.2012.05.007

[21] Gruver-Yates AL, Cidlowski JA. Tissue-specific actions of glucocorticoids on apoptosis: a double-edged sword. Cells. 2013;2: 202–23. doi: 10.3390/cells2020202 24709697 10.3390/cells2020202 PMC3972684

[22] De Bosscher K, Beck IM, Ratman D, Berghe W Vanden, Libert C. Activation of the Glucocorticoid Receptor in Acute Inflammation: the SEDIGRAM Concept. Trends in Pharmacological Sciences. 2016;37: 4–16. doi: 10.1016/j.tips.2015.09.002 26603477 10.1016/j.tips.2015.09.002

[23] De Bosscher K, Beck IM, Haegeman G. Classic glucocorticoids versus non-steroidal glucocorticoid receptor modulators: survival of the fittest regulator of the immune system? Brain, Behavior, and Immunity. 2010;24: 1035–1042. doi: 10.1016/j.bbi.2010.06.010 20600811 10.1016/j.bbi.2010.06.010

[24] Vettorazzi S, Bode C, Dejager L, Frappart L, Shelest E, Klaßen C, et al Glucocorticoids limit acute lung inflammation in concert with inflammatory stimuli by induction of SphK1. Nature Communications. 2015;6: 7796 doi: 10.1038/ncomms8796 26183376 10.1038/ncomms8796 PMC4518295

[25] De Bosscher K. Selective Glucocorticoid Receptor modulators. Journal of Steroid Biochemistry and Molecular Biology. 2010;120: 96–104. doi: 10.1016/j.jsbmb.2010.02.027 20206690 10.1016/j.jsbmb.2010.02.027

[26] Louw A, Swart P, de Kock SS, van der Merwe KJ. Mechanism for the Stabilization in. Viva of the Aziridine Precursor 2- (4-Acetoxyphenyl)-2-chloro-N-methyl-ethylammonium Chloride by Serum Proteins. Biochemical pharmacology. 1997;53: 189–197. 9037251 10.1016/s0006-2952(96)00661-2

[27] De Bosscher K, Berghe W V, Beck IME, Van Molle W, Hennuyer N, Hapgood J, et al A fully dissociated compound of plant origin for inflammatory gene repression. Proceedings of the National Academy of Sciences of the United States of America. 2005;102: 15827–15832. doi: 10.1073/pnas.0505554102 16243974 10.1073/pnas.0505554102 PMC1276063

[28] Yemelyanov A, Czwornog J, Gera L, Joshi S, Chatterton RT Jr., Budunova I. Novel steroid receptor phyto-modulator compound a inhibits growth and survival of prostate cancer cells. Cancer Research. 2008;68: 4763–4773. doi: 10.1158/0008-5472.CAN-07-6104 18559523 10.1158/0008-5472.CAN-07-6104

[29] Desmet SJ, Bougarne N, Van Moortel L, De Cauwer L, Thommis J, Vuylsteke M, et al Compound A influences gene regulation of the Dexamethasone-activated glucocorticoid receptor by alternative cofactor recruitment. Scientific Reports. 2017;7: 8063 doi: 10.1038/s41598-017-07941-y 28808239 10.1038/s41598-017-07941-y PMC5556032

[30] Dewint P, Gossye V, De Bosscher K, Vanden Berghe W, Van Beneden K, Deforce D, et al A plant-derived ligand favoring monomeric glucocorticoid receptor conformation with impaired transactivation potential attenuates collagen-induced arthritis. Journal of Immunology. 2008;180: 2608–2615. doi: 10.1136/ard.2008.102871 18250472 10.4049/jimmunol.180.4.2608

[31] Wüst S, Tischner D, John M, Tuckermann JP, Menzfeld C, Hanisch UK, et al Therapeutic and adverse effects of a non-steroidal glucocorticoid receptor ligand in a mouse model of multiple sclerosis. PLoS ONE. 2009;4: e8202 doi: 10.1371/journal.pone.0008202 19997594 10.1371/journal.pone.0008202 PMC2781169

[32] Zhang Z, Zhang Z-Y, Schluesener HJ. Compound A, a Plant Origin Ligand of Glucocorticoid Receptors, Increases Regulatory T Cells and M2 Macrophages to Attenuate Experimental Autoimmune Neuritis with Reduced Side Effects. The Journal of Immunology. 2009;183: 3081–3091. doi: 10.4049/jimmunol.0901088 19675162 10.4049/jimmunol.0901088

[33] van Loo G, Sze M, Bougarne N, Praet J, Mc Guire C, Ullrich A, et al Antiinflammatory Properties of a Plant-Derived Nonsteroidal, Dissociated Glucocorticoid Receptor Modulator in Experimental Autoimmune Encephalomyelitis. Molecular Endocrinology. 2010;24: 310–322. doi: 10.1210/me.2009-0236 19965930 10.1210/me.2009-0236 PMC5428123

[34] Thiele S, Ziegler N, Tsourdi E, De Bosscher K, Tuckermann JP, Hofbauer LC, et al Selective glucocorticoid receptor modulation maintains bone mineral density in mice. Journal of Bone and Mineral Research. 2012;27: 2242–2250. doi: 10.1002/jbmr.1688 22714558 10.1002/jbmr.1688

[35] Reber LL, Daubeuf F, Plantinga M, De Cauwer L, Gerlo S, Waelput W, et al A dissociated glucocorticoid receptor modulator reduces airway hyperresponsiveness and inflammation in a mouse model of asthma. Journal of immunology. American Association of Immunologists; 2012;188: 3478–87. doi: 10.4049/jimmunol.1004227 22393156 10.4049/jimmunol.1004227

[36] Yemelyanov A, Bhalla P, Yang X, Ugolkov A, Iwadate K, Karseladze A, et al Differential targeting of androgen and glucocorticoid receptors induces ER stress and apoptosis in prostate cancer cells: a novel therapeutic modality. Cell Cycle. 2012;11: 395–406. doi: 10.4161/cc.11.2.18945 22223138 10.4161/cc.11.2.18945 PMC3356826

[37] Zheng Y, Ishiguro H, Ide H, Inoue S, Kashiwagi E, Kawahara T, et al Compound A Inhibits Bladder Cancer Growth Predominantly via Glucocorticoid Receptor Transrepression. Molecular Endocrinology. 2015;29: 1486–1497. doi: 10.1210/me.2015-1128 26322830 10.1210/me.2015-1128 PMC5414678

[38] Lesovaya EA, Yemelyanov AY, Kirsanov KI, Yakubovskaya MG, Budunova IV. Antitumor effect of non-steroid glucocorticoid receptor ligand CpdA on leukemia cell lines CEM and K562. Biochemistry (Mosc). 2011;76: 1242–1252. doi: 10.1134/S000629791111006X 22117551 10.1134/S000629791111006X

[39] Lesovaya E, Yemelyanov A, Kirsanov K, Popa A, Belitsky G, Yakubovskaya M, et al Combination of a selective activator of the glucocorticoid receptor Compound A with a proteasome inhibitor as a novel strategy for chemotherapy of hematologic malignancies. Cell Cycle. 2013;12: 133–144. doi: 10.4161/cc.23048 23255118 10.4161/cc.23048 PMC3570502

[40] Hellemans J, Mortier G, De Paepe A, Speleman F, Vandesompele J. qBase relative quantification framework and software for management and automated analysis of real-time quantitative PCR data. Genome biology. 2007;8: R19 doi: 10.1186/gb-2007-8-2-r19 17291332 10.1186/gb-2007-8-2-r19 PMC1852402

[41] Hellemans J, Vandesompele J. Selection of reliable reference genes for RT-qPCR analysis. Methods in Molecular Biology. 2014;1160: 19–26. doi: 10.1007/978-1-4939-0733-5_3 24740218 10.1007/978-1-4939-0733-5_3

[42] Robertson S, Allie-Reid F, Vanden Berghe W, Visser K, Binder A, Africander D, et al Abrogation of Glucocorticoid Receptor Dimerization Correlates with Dissociated Glucocorticoid Behavior of Compound A. Journal of Biological Chemistry. 2010;285: 8061–8075. doi: 10.1074/jbc.M109.087866 20037160 10.1074/jbc.M109.087866 PMC2832957

[43] Vandevyver S, Dejager L, Libert C. On the trail of the glucocorticoid receptor: into the nucleus and back. Traffic. 2012;13: 364–374. doi: 10.1111/j.1600-0854.2011.01288.x 21951602 10.1111/j.1600-0854.2011.01288.x

[44] Beck IM, Drebert ZJ, Hoya-Arias R, Bahar AA, Devos M, Clarisse D, et al Compound A, a Selective Glucocorticoid Receptor Modulator, Enhances Heat Shock Protein Hsp70 Gene Promoter Activation. PLoS ONE. 2013;8: e69115 doi: 10.1371/journal.pone.0069115 23935933 10.1371/journal.pone.0069115 PMC3728325

[45] Daugaard M, Rohde M, Jäättelä M. The heat shock protein 70 family: Highly homologous proteins with overlapping and distinct functions. FEBS Letters. 2007;581: 3702–3710. doi: 10.1016/j.febslet.2007.05.039 17544402 10.1016/j.febslet.2007.05.039

[46] Greenstein S, Krett NL, Kurosawa Y, Ma C, Chauhan D, Hideshima T, et al Characterization of the MM.1 human multiple myeloma (MM) cell lines: a model system to elucidate the characteristics, behavior, and signaling of steroid-sensitive and -resistant MM cells. Experimental Hematology. 2003;31: 271–282. doi: 10.1016/S0301-472X(03)00023-7 12691914 10.1016/s0301-472x(03)00023-7

[47] Medh RD, Webb MS, Miller AL, Johnson BH, Fofanov Y, Li T, et al Gene expression profile of human lymphoid CEM cells sensitive and resistant to glucocorticoid-evoked apoptosis. Genomics. 2003;81: 543–55. doi: 10.1016/S0888-7543(03)00045-4 12782123 10.1016/s0888-7543(03)00045-4 PMC2777808

[48] Deroo BJ, Archer TK. Glucocorticoid Receptor Activation of the IκBα Promoter within Chromatin. Molecular Biology of the Cell. 2001;12: 3365–3374. 11694573 10.1091/mbc.12.11.3365 PMC60261

[49] Altonsy MO, Sasse SK, Phang TL, Gerber AN. Context-dependent Cooperation between Nuclear Factor kB (NF-κB) and the Glucocorticoid Receptor at a TNFAIP3 Intronic Enhancer A MECHANISM TO MAINTAIN NEGATIVE FEEDBACK CONTROL OF INFLAMMATION. Journal of Biological Chemistry. 2014;289: 8231–8239. doi: 10.1074/jbc.M113.545178 24500711 10.1074/jbc.M113.545178 PMC3961651

[50] Rauner M, Goettsch C, Stein N, Thiele S, Bornhaeuser M, De Bosscher K, et al Dissociation of osteogenic and immunological effects by the selective glucocorticoid receptor agonist, compound A, in human bone marrow stromal cells. Endocrinology. 2011;152: 103–112. doi: 10.1210/en.2010-0456 21084452 10.1210/en.2010-0456

[51] Robertson S, Hapgood JP, Louw A. Glucocorticoid receptor concentration and the ability to dimerize influence nuclear translocation and distribution. Steroids. 2013;78: 182–194. doi: 10.1016/j.steroids.2012.10.016 23178279 10.1016/j.steroids.2012.10.016

[52] Drebert Z, Bracke M, Beck IM. Glucocorticoids and the non-steroidal selective glucocorticoid receptor modulator, compound A, differentially affect colon cancer-derived myofibroblasts. The Journal of steroid biochemistry and molecular biology. 2015;149: 92–105. doi: 10.1016/j.jsbmb.2015.02.002 25666906 10.1016/j.jsbmb.2015.02.002

[53] Ronacher K, Hadley K, Avenant C, Stubsrud E, Simons SS, Louw A, et al Ligand-selective transactivation and transrepression via the glucocorticoid receptor: Role of cofactor interaction. Molecular and Cellular Endocrinology. 2009;299: 219–231. doi: 10.1016/j.mce.2008.10.008 19007848 10.1016/j.mce.2008.10.008

[54] Clarisse D, Thommis J, Van Wesemael K, Houtman R, Ratman D, Tavernier J, et al Coregulator profiling of the glucocorticoid receptor in lymphoid malignancies. Oncotarget. 2017;8: 109675–109691. doi: 10.18632/oncotarget.22764 29312638 10.18632/oncotarget.22764 PMC5752551

[55] Gavrila A, Chachi L, Tliba O, Brightling C, Amrani Y. Effect of the plant derivative compound a on the production of corticosteroid-resistant chemokines in airway smooth muscle cells. American Journal of Respiratory Cell and Molecular Biology. 2015;53: 728–737. doi: 10.1165/rcmb.2014-0477OC 25897650 10.1165/rcmb.2014-0477OC PMC4742953

[56] Meijsing SH, Pufall MA, So AY, Bates DL, Chen L, Yamamoto KR. DNA Binding Site Sequence Directs Glucocorticoid Receptor Structure and Activity. Science. 2009;324: 407–410. doi: 10.1126/science.1164265 19372434 10.1126/science.1164265 PMC2777810

[57] Schmidt S, Rainer J, Ploner C, Presul E, Riml S, Kofler R. Glucocorticoid-induced apoptosis and glucocorticoid resistance: molecular mechanisms and clinical relevance. Cell Death and Differentation. 2004;11: S45–55. doi: 10.1038/sj.cdd.4401456 15243581 10.1038/sj.cdd.4401456

[58] Greenstein S, Ghias K, Krett NL, Rosen ST. Mechanisms of glucocorticoid-mediated apoptosis in hematological malignancies. Clinical Cancer Research. 2002;8: 1681–1694. 12060604

[59] Schaaf MJ, Cidlowski JA. Molecular mechanisms of glucocorticoid action and resistance. Journal of Steroid Biochemistry & Molecular Biology. 2003;83: 37–48. doi: 10.1016/S0960-0760(02)00263-7 10.1016/s0960-0760(02)00263-7 12650700

[60] Chen Z, Lan X, Wu D, Sunkel B, Ye Z, Huang J, et al Ligand-dependent genomic function of glucocorticoid receptor in triple-negative breast cancer. Nature communications. 2015;6: 8323 doi: 10.1038/ncomms9323 26374485 10.1038/ncomms9323 PMC4573460

