# Supplementary material for: Effect of combining glucocorticoids with Compound A on glucocorticoid receptor responsiveness in lymphoid malignancies
Source: PLoS One. 2018 May 8;13(5):e0197000. doi: 10.1371/journal.pone.0197000 (PMC5940183; doi:10.1371/journal.pone.0197000)
Supplement: S1 Table — (DOCX) [file pone.0197000.s001.docx]

| **Target** | **Primer forward** | **Primer reverse** |
| --- | --- | --- |
| HSPA1A | AGGTGCAGGTGAGCTACAAGG | GGTCAGCACCATGGACGAG |
| FKBP5 | AGTAGAAATCCACCTGGAAGGC | ATTTAGGCTTCCCTGCCTCT |
| GILZ | GCGTGAGAACACCCTGTTGA | TCAGACAGGACTGGAACTTCTCC |
| GR | TGATGAAGCTTCAGGATGTCA | TTCGAGCTTCCAGGTTCATTC |
| A20 | CCTTGCTTTGAGTCAGGCTGT | AAGGAGAAGCACGAAACATC |
| IκBα | CTCCGAGACTTTCGAGGAAATAC | GCCATTGTAGTTGGTAGCCTTCA |
| RANTES | TGCCCACATCAAGGAGTATTT | CTTTCGGGTGACAAAGACG |
| ICAM | GCAGACAGTGACCATCTACAGCTT | CTTCTGAGACCTCTGGCTTCGT |
| RPL13A | CCTGGAGGAGAAGAGGAAAGAGA | TTGAGGACCTCTGTGTATTTGTCAA |
| SDHA | TGGGAACAAGAGGGCATCTG | CCACCACTGCATCAAATTCATG |
| YWHAZ | ACTTTTGGTACATTGTGGCTTCAA | CCGCCAGGACAAACCAGTAT |
