# Supplementary figures and images for: Effect of combining glucocorticoids with Compound A on glucocorticoid receptor responsiveness in lymphoid malignancies
Source: PLoS One. 2018 May 8;13(5):e0197000. doi: 10.1371/journal.pone.0197000 (PMC5940183; doi:10.1371/journal.pone.0197000)

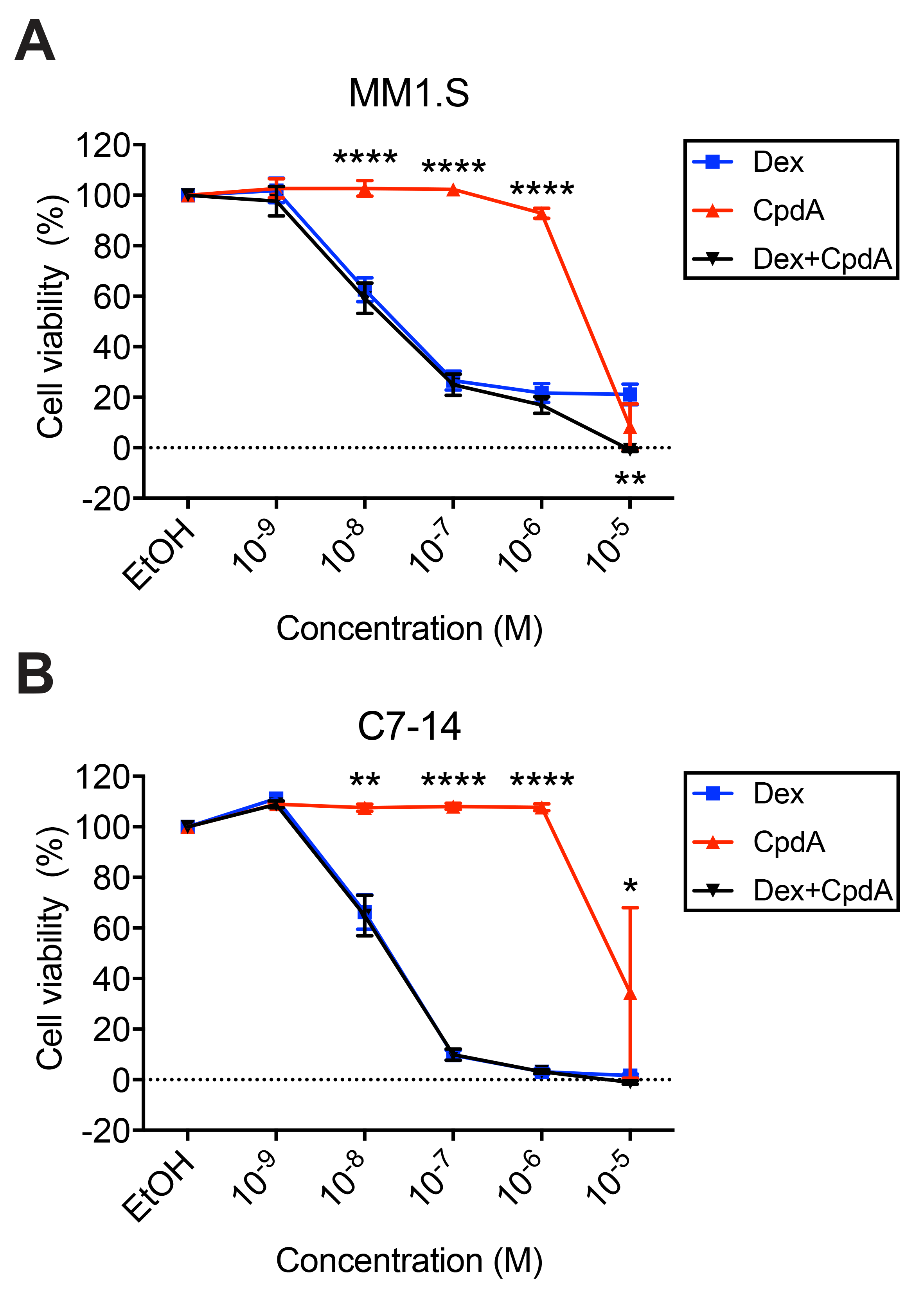

Supplement: S1 Fig — (A) MM1.S (MM) and (B) C7-14 (ALL) cells were treated for 72h with a concentration range (10-5M-10-9M) of Dex, CpdA or Dex/CpdA combination (equimolar concentrations). The cell viability was determined using CellTiter-Glo assays. The cell viability of the solvent control (EtOH) was set at 100% and all other cell viabilities were normalized accordingly. The scatter plots represent the mean +/- SEM of 3 independent experiments. Statistical analysis was performed using GraphPad Prism 7, using a two-way ANOVA with Tukey’s multiple comparison post-test, comparing Dex vs. CpdA or vs. Dex/CpdA per concentration. Only significant differences are displayed: * = P < 0.05, ** = P < 0.01, **** = P < 0.0001. (TIF) [file pone.0197000.s002.tif]

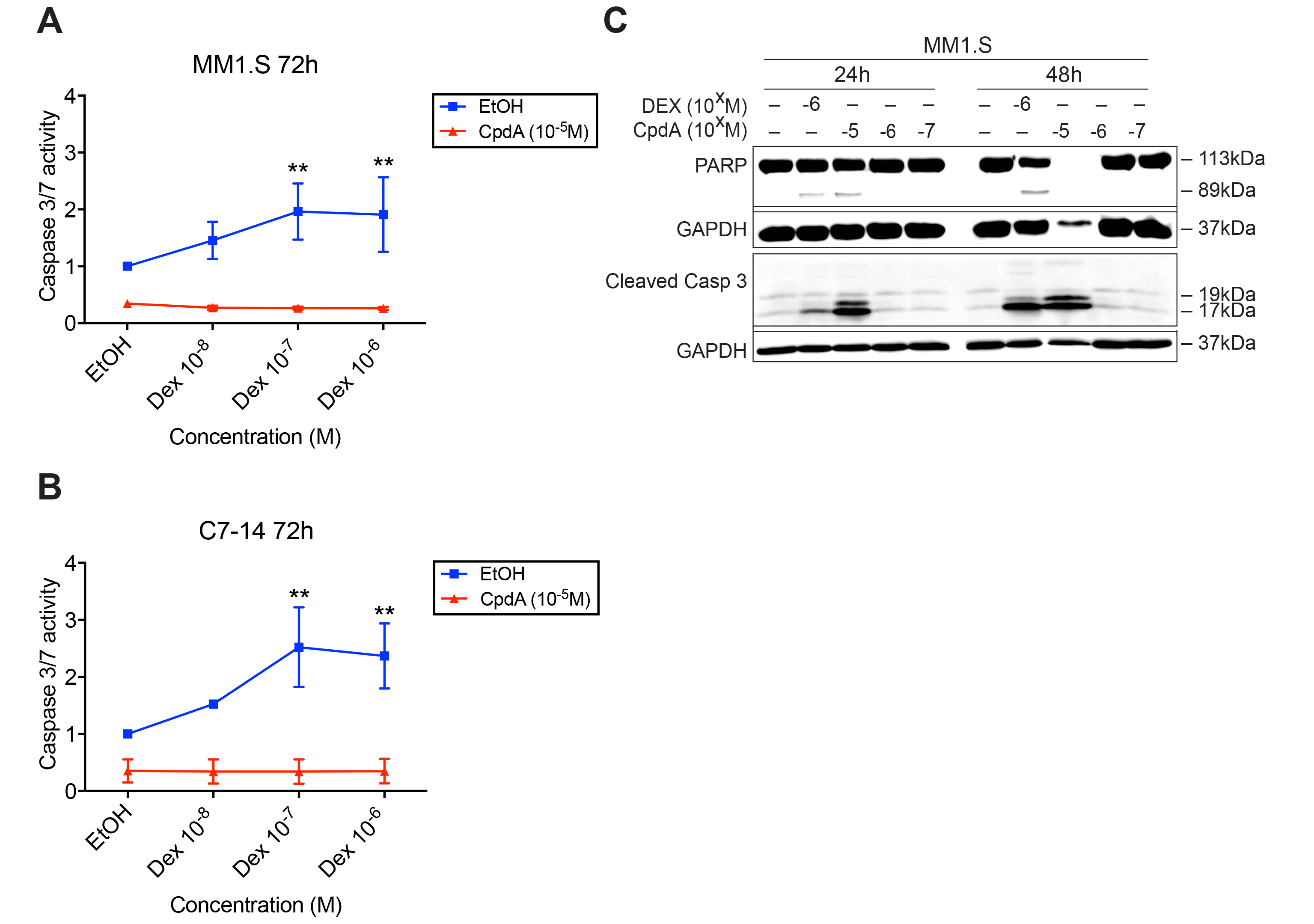

Supplement: S2 Fig — (A) MM1.S and (B) C7-14 cells were treated for 72h with a Dex concentration range (10-6M-10-8M), CpdA (10μM) or Dex/CpdA combination (fixed CpdA concentration). The caspase activity was determined using Caspase-Glo 3/7 assays. The caspase 3/7 activity of the solvent control (EtOH) was set at 1 and all other values were normalized accordingly. The scatter plots represent the mean +/- SEM of 3 independent experiments. Statistical analysis was performed using GraphPad Prism 7, using a two-way ANOVA with Sidak’s multiple comparison post-test, comparing Dex vs. Dex/CpdA per concentration. Only significant differences are displayed: ** = P < 0.01. (C) MM1.S cells were treated for 24h or 48h with solvent, 10-6M Dex or a limited CpdA concentration range (10-5M-10-7M). Protein lysates were subjected to WB analysis, determining the protein levels of PARP (89 and 113kDa) and cleaved-caspase 3 (17-19kDa), with GAPDH (37kDa) serving as loading control. Results are representative of 3 independent experiments. (TIF) [file pone.0197000.s003.tif]

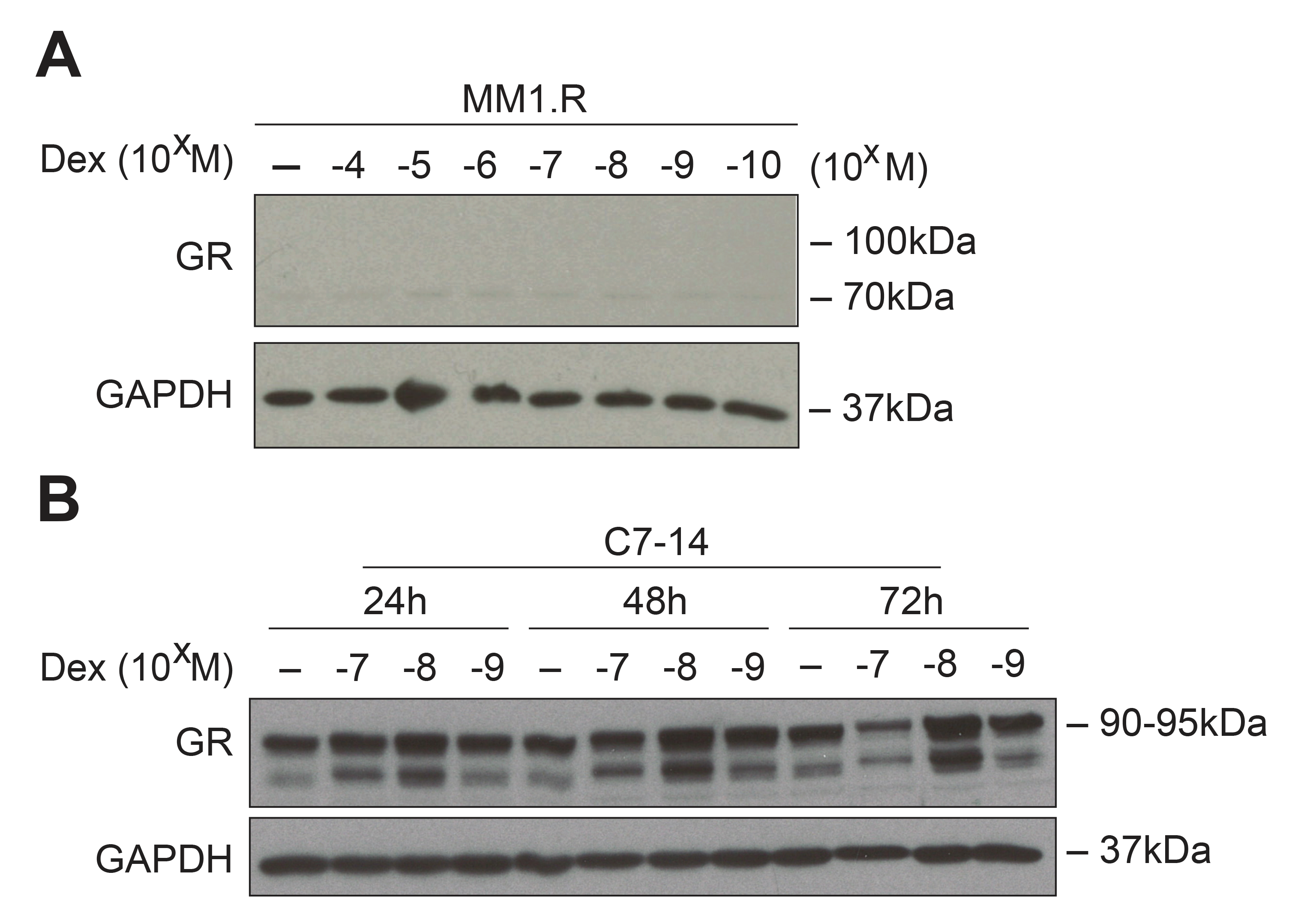

Supplement: S3 Fig — (A) MM1.R cells were treated for 72h with a Dex concentration range (10-4M-10-10M). (B) C7-14 cells were treated for 24h, 48h or 72h with a Dex concentration range (10-7M-10-9M). (A-B) Protein lysates were prepared and WB analysis was performed, detecting the protein levels of GR (90-95kDa), with GAPDH (37kDa) serving as loading control. WB results arise from 1 (A) biological experiment, or are representative of 2 (B) biological experiments. (TIF) [file pone.0197000.s004.tif]

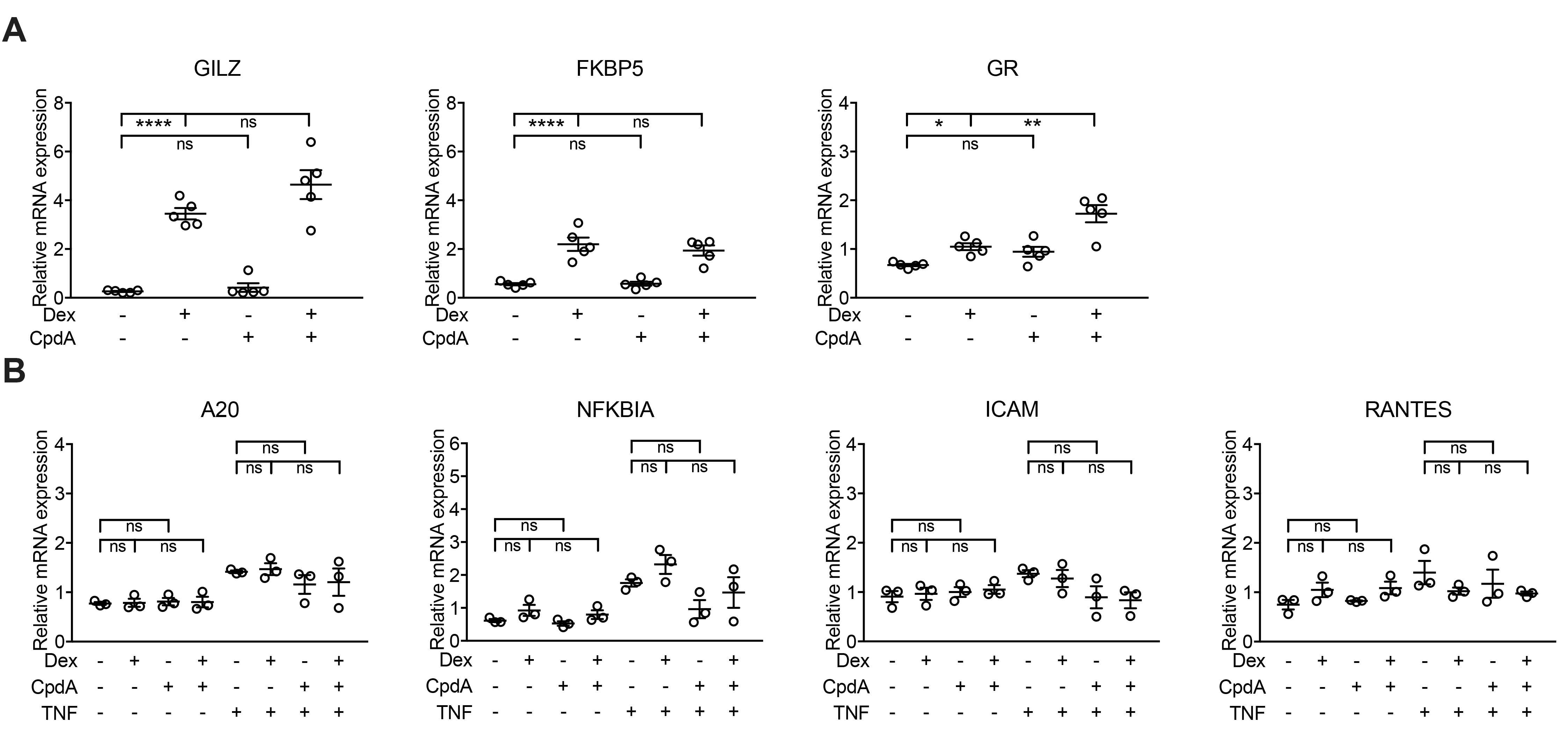

Supplement: S4 Fig — C1-15 cells were treated for 6h with Dex (1μM), CpdA (10μM) or Dex/CpdA combination. RNA was isolated and subjected to RT-QPCR, detecting the mRNA levels of (A) GILZ, FKBP5 and GR as a measure for transactivation and (B) A20, NFKBIA, ICAM and RANTES as a measure for transrepression. (A-B) SDHA, YWHAZ and RPL13A served as reference genes. The dot plots represent the mean +/- SEM of 5 (A) or 3 (B) biological replicates with the open circles (o) representing the mean of each biological experiment. A two-way ANOVA with Tukey’s multiple comparison post-test was performed on log transformed data using GraphPad Prism 7. * = P < 0.05, ** = P < 0.01, **** = P < 0.0001, ns = non-significant. (TIF) [file pone.0197000.s005.tif]
